# High-resolution multi-modal imaging of sub-cellular structures with low numerical aperture objective

**DOI:** 10.1088/2515-7647/adc04f

**Published:** 2025-03-25

**Authors:** Somaiyeh Khoubafarin, Peuli Nath, Saloni Malla, Durgesh Desai, William D Gorgas, Amit K Tiwari, Aniruddha Ray

**Affiliations:** 1 Department of Physics and Astronomy, University of Toledo, Toledo, OH 43606, United States of America; 2 Department of Pharmacology and Experimental Therapeutics, University of Toledo, Toledo, OH 43606, United States of America; 3 Department of Pharmaceutical Sciences, College of Pharmacy, University of Arkansas of Medical Sciences, Little Rock, AR, United States of America; 4 Department of Electrical and Computer Science, University of Toledo, Toledo, OH 43606, United States of America

**Keywords:** microlens substrate, resolution, field of view

## Abstract

Imaging of subcellular structures, which underpins many of the advances in biological and medical sciences, requires microscopes with high numerical aperture (N.A.) objectives which are costly, complex, requires oil immersion and have very limited field-of-view, typically covering a handful of cells. Here, we leverage a low N.A. objective to simultaneously capture scattering, phase, and fluorescence images of subcellular structures in breast cancer cells (BT-20) and observe nanoparticle uptake, with sub-diffraction-limited resolution (<400 nm with a 0.25 N.A. objective) utilizing a 2-dimensional (2-D) microlens substrate. High resolution labeled and label-free images of subcellular components is made possible by implementing a specific configuration, wherein the sample is placed in close proximity to the microlens substrate, which results in efficient collection of the rapidly decaying evanescent waves that contains the high frequency information, thereby improving resolution and the light capture efficiency. The microlens-assisted imaging provides an easy-to-implement and cost-effective means of drastically improving the resolution of any microscope with low N.A. objective lenses, paving the way for the development of affordable, portable multi-modal imaging systems with high-resolution imaging capabilities. This technology has broad implications for various fields and could democratize access to high-quality microscopy, particularly for application in resource-limited settings.

## 1 Introduction

Numerical aperture (N.A.) of objective lenses is one of the most important parameters for optical microscopy, as it affects the lens’s light-collection ability and, governs the image resolution, signal to noise ratio (SNR) and field of view [1]. For any objective lens, there is a trade-off between the resolution and field-of-view (FOV), due to finite space-bandwidth product. While high NA objective facilitates high resolution and high SNR, enabling imaging of nanostructures within biological samples, they have a very limited FOV. Additionally, they are costly and difficult to use. Whereas low NA objectives are cheap, easy-to use and enables imaging over a wide field of view, which makes them particularly attractive for portable microscopy applications. However, they suffer from low resolution and SNR, resulting in inferior image quality and inability to observe nanoscale structures, such as cell organelles [2].

Several different strategies have been implemented over the years to overcome the aforementioned limitations related to image resolution by using techniques such as pixel-super resolution (PSR) [3–6], Fourier ptychography (FP) [7–10], structured illumination microscopy (SIM) [11–13]. However, some of these techniques are time intensive due to the requirement of acquiring multiple images and costly due to requirement of additional hardware (e.g. spatial light modulators for SIM and piezo stages for PSR and FP). Additionally, computational techniques including digital deconvolution, and deep learning frameworks have also been implemented [14, 15]. Implementing digital deconvolution requires technical expertise and deep learning approaches can be computationally intensive. Furthermore, new sample types necessitate fresh training for each instance.

Here, we utilize a 2-dimensional microlens substrate assisted imaging technique, achieving a threefold enhancement in resolution, and resulting in sub-diffraction limited images. This method uniquely employs microlens substrate which are placed in contact with the sample to form a sandwich structure. The resolution is enhanced as a result of virtual image formation by individual microlens, a phenomenon confirmed through finite-difference time-domain (FDTD) simulations. This reusable substrate, containing the microlens substrate, can be effortlessly affixed to and removed from the sample, causing minimal to no damage. Although microlenses have been previously used to improve image resolution [16–26], only a handful were used for biological applications [27–31]. Additionally, all of these studies used high N.A. objectives (Magnification >40×), mostly with oil immersion, in order to observe subcellular structures.

Our approach improves the resolution and light collection of low NA (∼0.25) objective lenses in multiple-modalities by utilizing microlens substrate close to the sample. We investigated a range of microlens with diameters ranging from 40 to 500 *μ*m and refractive indices from ∼1.4 to 2 to image fluorescent nanoparticles (46 nm −1 *μ*m). We achieved the best results using microlenses with diameter of ∼150 *μ*m and a refractive index of 1.48, as this allowed us to transcend conventional resolution limits and obtain sub-400 nm resolution. The microlens substrates were implemented in both label-free methods such as dark field and phase contrast microscopy and labeled methods such as fluorescence microscopy. Using this technique, we were able to image breast cancer cells internalized with fluorescent nanoparticles, capturing subcellular structures such as endosomes/lysosomes and chromosomes merely using a 10× objective. This method can have far-reaching implications, as it can upgrade any low-resolution microscope to a high-resolution version. This will be impactful for both medical diagnostics, basic science research as well as student training, particularly using portable/mobile microscopes in resource-limited areas.

## 2 Materials and method

### 2.1 Materials

Borosilicate solid glass microlens with refractive index of 1.48 was purchased from Cospheric (Santa Barbara, CA, US). Copolymer microlens suspensions and green fluorescence polymer particles with the size of 46 nm, 200 nm, and 1 *μ*m was purchased from Thermo scientific. Cesium bromide (CsBr), Lead bromide (PbBr2), Oleylamine (OAm), Oleic acid (OA), Tetramethyl orthosilicate (TMOS), (3-aminopropyl) Triethoxy silane 99% (APTES) were all acquired from Sigma-Aldrich (St. Louis, MO). Ammonia hydroxide, N’, N, dimethylformamide, toluene (0.006% water content) was obtained from Fisher Chemical. RPMI 1640 media, DMEM (without phenol red), DAPI ((4′,6-diamidino-2-phenylindole) and other cell culture reagents were obtained from Invitrogen. All solutions were prepared in ACS reagent grade water. All the chemicals and materials were used as received.

### 2.2 Preparation of microlenses

**Figure 1:**
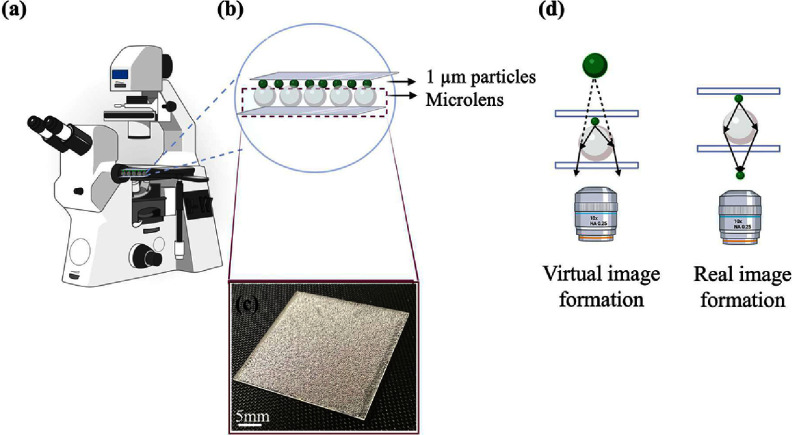
Schematic view of (a) Multimodal microscope, (b) Sandwich structure of sample and microlens. (c) Image of microlens substrate captured by a mobile phone. (d) Formation of real and virtual images.

We evenly dispersed a variety of microlens on a glass coverslip to create a monolayer substrate of microlenses. The microlens used included dry borosilicate solid glass with a diameter of 150–180 *μ*m and a refractive index of 1.48, glass beads with a diameter of 0.5 mm and a refractive index of 1.5, as well as a polymeric microlens with a refractive index of 1.59 and a diameter of 42 *μ*m suspended in water. Image of microlens substrate captured by mobile phone is illustrated in figure 1(c).

### 2.3 Imaging of fluorescent particle

A multimodal inverted microscope (LEICA DMi8) with dark field, phase contrast, and fluorescence modalities was used to investigate microlens-assisted imaging resolution. The dark field and phase contrast modalities (white light illumination with wavelength in the range of 400–700 nm) were transmission-based. The fluorescence microscope setup included DAPI (Ex: 360 nm, and Em_max_: 460 nm), FITC (Ex. 495 nm and Em_max_: 520 nm), and TXR (Ex: 595 nm, and Em_max_: 615 nm) filters. For imaging nanoparticles within cells, the FITC channel was used, and for imaging the nucleus of the cells, the DAPI channel was utilized. Imaging was conducted with a 10X objective (NA of 0.25), using a Leica DFC3000 G camera with a 1.3-megapixel CCD monochrome sensor.

We imaged fluorescently labeled polystyrene particles with sizes ranging from 46 nm to 1 *μ*m, A hydrophilic surface was created using a handheld plasma generator to minimize particle aggregation, followed by air drying. We performed imaging with and without microlens to assess resolution enhancement. The schematic image of set-up was shown in figure 1(a). The microlens substrate and polystyrene particles formed a sandwich structure, as shown in figure 1(b). Scanning electron microscopy (SEM) was also used to compare resolution, with all SEM images captured using HITACHI S-4800.

### 2.4 Imaging nano particle uptake in breast cancer cell

We imaged breast cancer cells treated with Silica coated Perovskite quantum dots (PQDs@SiO2) using microlens-assisted optical microscope. We synthesized silica coated perovskite quantum dots by following a two-step modified method for the synthesis procedure previously reported [32, 33].

a **Synthesis of precursor solution-** First we prepared a precursor solution, 0.1468 g of PbBr2, 0.0851 g of CsBr, 0.6 ml of OAm, 1.8 ml of OA was added to 10 ml of N’, N’ dimethyl formamide under constant stirring condition at 90 °C until a clear solution was obtained.
b **Synthesis of PQDs@SiO2**- We added 40 *μ*l of 2.8% ammonia hydroxide to the 2 ml of the precursor solution prepared in the above procedure. For one-pot synthesis, 0.2 ml of the precursor solution + ammonia was quickly added into 10 ml of dry toluene containing 5 *μ*l TMOS under vigorous stirring at 1500 rpm. After 10 sec, the stirring speed was adjusted to 350 rpm and kept for 8 h at 30 °C to complete the reaction. The final product PQDs@SiO2 nanoparticle were collected in the form of pellet by centrifugation at 9000 rpm for 10 min and dried in oven at 50 °C for further use.
c **Modification of PQDs@SiO2 nanoparticles for intracellular studies**- We dispersed approx. 4 mg of the dried PQDs@SiO2 nanoparticles in 500 *μ*l absolute ethanol solution using sonication. For the functionalization of the nanoparticle surface with amino group, we added 20 *μ*l of APTES to the nanoparticle solution and stirred for 15 min. After that, the modified nanoparticle mixture solution was centrifuged at 9000 rpm for 10 min and washed with deionized water 4 times to remove any traces of ethanol and finally dispersed in 500 *μ*l phosphate buffer for the cell experiment.
d **Internalization of PQDs@SiO2 nanoparticle inside live cells**- The BT-20 cells were cultured in 10% serum-supplemented RPMI-1640 cell medium. For microscopy experiments the cells were cultured on a glass coverslip and experiment was performed when the cells reached 60% confluency. The cells were washed thoroughly with phosphate buffer solution and 2 ml colourless DMEM media was added with concentrated nanoparticle solution (200 *μ*l). The cells were incubated with the nanoparticle solution for 1 h at 37 °C in 5% CO2 environment. After the incubation step, cells were washed with PBS solution 3 times to remove any excess nanoparticle. To confirm the internalization, the cells with nanoparticles were imaged under fluorescence microscope using FITC at 10X magnification.
e **Fixing and staining of cells**- The cells with internalized PQDs@SiO2 nanoparticle was fixed using 4% paraformaldehyde. 1 ml of 4% PFA was added to cover the entire area of the cover slip and incubated for 15 min at room temperature. After 15 min, the cells were washed thoroughly with PBS solution. Next, the nucleus of the cells was stained with DAPI, 1 ml DAPI (300 nM) solution was added to cover slip containing the fixed cells and incubated in dark for 8 min. After 8 min, the cells were washed with PBS to remove any excess stain, and fixed cells were kept in 8 °C for further experiment. The stained cells were imaged under fluorescence microscope using DAPI at 10× magnification.

## 3 Result and discussion

### 3.1 The mechanism of image formation using microlens

Conventional optical microscopes have limited resolution which is dictated by the diffraction limit as images are formed by focusing the propagating wave that stems from the sample [34, 35]. Microlens-assisted techniques, however, circumvent this barrier using three distinct mechanisms: the formation of enlarged virtual images, photonic nano-jets, and the conversion of evanescent waves into propagating waves for enhanced detail capture [36–40].

**Figure 2:**
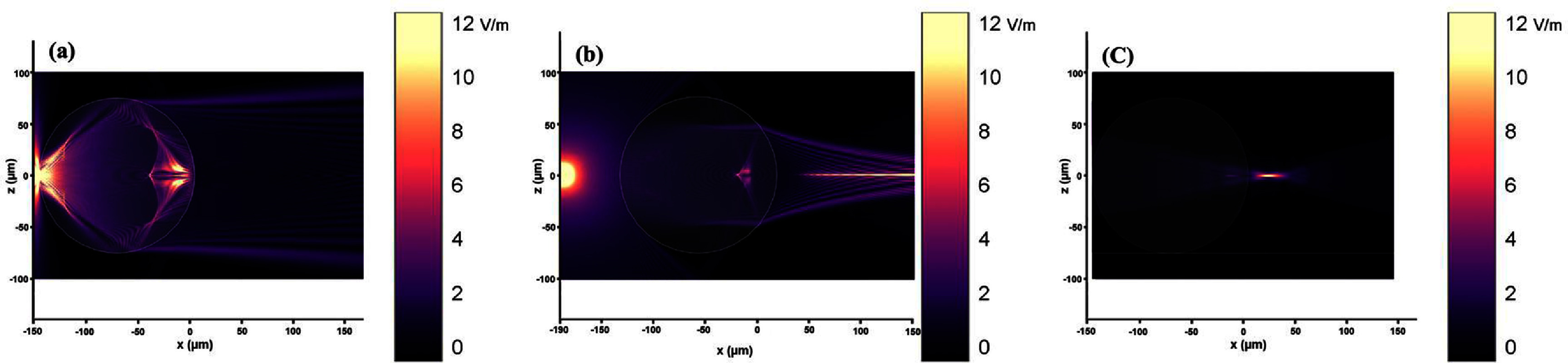
2D simulation using FDTD, displaying Electric field vector for the microlens with refractive index of 1.48 and diameter of 150 *μ*m. (a) A dipole excitation is located 2 *μ*m far from the left surface of microlens. (b) The dipole is 15 *μ*m away from the left microlens’s focal point. (c) Formation of photonic nanojet upon illumination of microlens from left side with plane wave.

Image formation using microlens vary depending on several factors, including microlens size, refractive index, and the distance from the object being imaged [22]. As shown in figure 1(d), if the object is located out of the focal length of the microlens, a real image is formed. It is smaller than the object and inverted. However, if the object is within the microlens’ focal length, an enlarged virtual image is formed that is in the same orientation as the object [39, 41, 42]. This was also validated using FDTD simulations (Flexcompute’s Tidy3D FDTD platform) in two dimensions. The simulations involved placing a TE mode dipole excitation close to the surface of a microlens (within the focal length of microlens) with diameter of 150 *μ*m and refractive index of 1.48. This results in diverging beam, indicating the formation of a virtual image as shown in figure 2(a). In the second simulation, positioning the dipole 15 *μ*m away from microlens’s focal point (which is ∼40.6 *μ*m) resulted in the convergence of electric field rays atop the microlens, thereby generating a real image spot depicted in figure 2(b). Furthermore, figure 2(c) is illustrating the formation of photonic nanojet.

**Figure 3:**
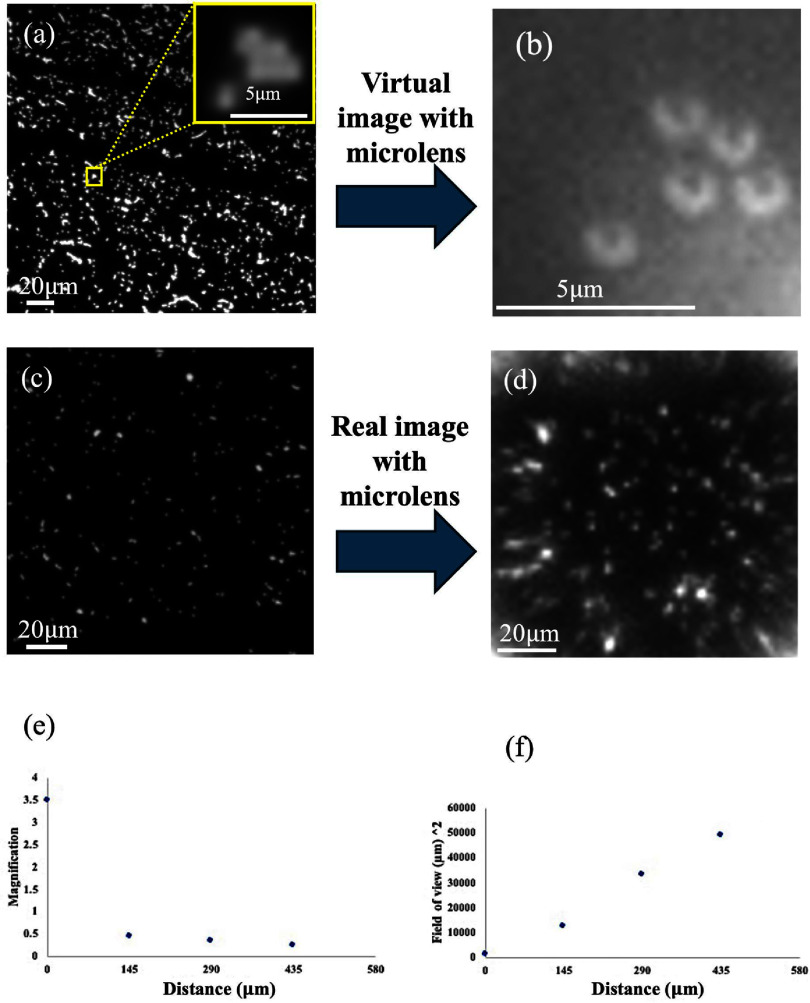
Image of 1 *μ*m fluorescent particles taken using 10× objective with and without the microlens. First row: virtual image configuration (a) without microlens (b) with microlens. Second row: real image configuration: (c) without microlens (d) with microlens. (e) The changes in image magnification as a function of microlens-sample distance. (f) Change in field of view of individual microlens as a function of microlens-sample distance. The distance between fluorescent particles and microlens is changed by adding coverslips between them.

Figure 3 illustrate the formation of virtual and real images using microlens substrate. The image of particles acquired without microlens is shown in figure 3(a). The virtual image of the particles within the region of interest (figure 3(a) inset) is presented in figure 3(b). The virtual image was generated by placing the object and the microlens substrate in close contact, ensuring the object was within the focal length of the microlens. This arrangement was achieved using a sandwich structure between the sample and the microlens substrate. A comparison of figure 3(a) (inset) and (b) highlights an improvement in resolution due to the formation of the virtual image. Furthermore, we also explored the formation of real images by placing multiple coverslips between the object and microlens to position the object beyond the focal length of the microlens. The real image of the sample acquired without and with using the microlens is shown in figures 3(c) and (d) respectively. The real image obtained in figure 3(d) was initially inverted which is a characteristic feature of real image formation; however, it was rotated to maintain the same orientation as the image without the microlens shown in figure 3(c). The original image is provided in the supplementary section (figure S2). Comparing figures 3(b) and (d) reveals that real image formed using the microlens has smaller magnification compared to the virtual image but offers a broader field of view, allowing a single microlens to cover a wider area. Further imaging was conducted to investigate changes in magnification and field of view for a single microlens under different conditions: with the object and microlens in close contact (sandwich structure), and with one, two, and three coverslips (each approximately ∼100–150 *μ*m thick) added between them. The resulting magnification and field of view measurements were plotted, as shown in figures 3(e) and (f), respectively. Here we focused on the field of view of a single microlens to achieve better clarity and observe detailed features, as the full-field image from the microlens substrate lacked sufficient resolution. The virtual images provide higher resolution (magnification) but at the cost of a reduced field of view. This limitation can be partially addressed by capturing multiple images while shifting the microlens substrate.

Furthermore, this approach uniquely enables imaging across a wide field of view with two different magnifications by adjusting the focal height. By changing the focus, it is possible to visualize enlarged virtual images formed by the microlenses as well as images of the sample positioned between the microlenses using the original objective. As shown in figure S1, the microlenses generate enlarged virtual images at their focal points, while regions outside the microlenses can be observed using the original 10× objective by simply altering the focal height.

### 3.2 Imaging polystyrene particles in multimodal microscopy

Broadly optical microscopy can be categorized into two general classes: label-free and labelled [43–45]. Label-free imaging techniques, such as phase contrast and dark field microscopy, enable samples to be visualized without the need for additional agents, revealing details about the innate characteristics of the specimens [46]. However, labeled imaging usually provides better contrast and specificity by highlighting certain components of interest within the sample using tags or stains, e.g. fluorescent dyes [47]. This section investigates the use of microlens-assisted imaging technique for both labeled and label-free imaging modalities in order to substantially improve image resolution.

To evaluate the microlens-assisted optical imaging technique, we characterized its performance using fluorescently labeled polystyrene particles of varying sizes, ranging from 46 nm to 1 *μ*m, across different imaging modalities, using microlens of various sizes and refractive indices, ranging from 42 *μ*m to 0.5 mm. Smaller lenses were initially tested but posed difficulties due to their short focal length which made it challenging to keep the sample within the focal length distance. To overcome this, larger microlens were considered; however, they lacked stability in our experimental arrangement even if they had larger focal length. Focal length and its dependence on diameter and refractive index of microlens were shown in supplementary section (S6). After a series of experiments, we selected dry borosilicate solid glass microlens with diameters of 150–180 *μ*m and a refractive index of 1.48. These were not only stable on the coverslip, but they also featured a sufficiently large focal length, ensuring the sample remained within the focal plane. Furthermore, the use of microlenses with a lower refractive index less than 2 circumvented the need for oil immersion, greatly simplifying the imaging process. Furthermore, low refractive index (*n*) microlens exhibit lower spherical and chromatic aberrations, compared to the high-*n* ones with similar dimensions. However, the aberrations in high-*n* microlenses can be mitigated using index-matching oil, though this significantly complicates the imaging process.

**Figure 4:**
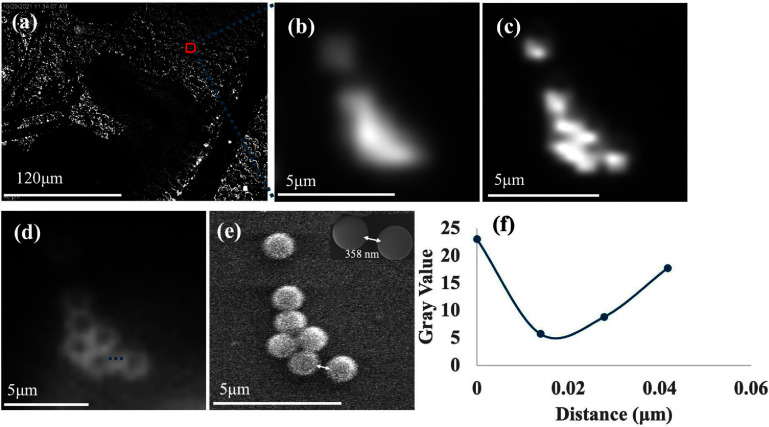
Dark field image of 1 *µ*m fluorescently labeled polystyrene particles taken by (a) a 10× objective with NA of 0.25. (b) Magnified image of area of interest without microlens using a 10× objective with NA of 0.25. (c) Magnified image of area of interest without microlens using 20× objective with an NA of 0.46. (d) Using 150–180 *µ*m microlens and a 10× objective with NA of 0.25. (e) SEM images of a 1 *µ*m fluorescent particle. (e) Light intensity profile along the dashed line shown in (e). Here the background intensity was subtracted from the signal.

The magnification achieved using microlens substrate is shown in figure 4. These images were acquired using dark field microscopy, which is a label-free technique that harnesses an oblique beam of light to illuminate unstained samples generating images from the scattered photons. The microlens enables imaging with resolution of sub-400 nm, which is beyond the diffraction limit of the 10× objective. This was estimated by measuring the distance between the 1 *μ*m particles that can be clearly resolved using the microlens substrate, as shown in figure 4. The full field of view image acquired using a 10× objective is shown in figure 4(a), followed by a magnified region of interest in figure 4(b). Figure 4(c) depicts the image acquired using 20× objective, whereas figure 4(d) illustrates the image captured with a 10× objective in the presence of microlens. The corresponding SEM image is shown in figure 4(e). The spatial variation of the intensity in between the two 1 *μ*m particles (along the dashed line shown in figure 4(d)) is shown in figure 4(f). We further verified this using a super high-resolution USAF test target (Newport), using phase contrast modality, as shown in figure S4. Achieving a sub-400 nm resolution indicates that our microlens-assisted system significantly improves the resolution of low NA objective, allowing the observation of finer details beyond the diffraction-limited capabilities of the conventional setup. Using the microlens provided ∼4× magnification, enabling clear distinction of individual polystyrene particles. In contrast, the individual particles cannot be distinguished when using just the 10× and 20× objectives with N.A. of 0.25 and 0.40 respectively. This result demonstrates the significant impact of microlens on the resolution of microscopy systems, which will make it possible to image structures that are too small to be resolved.

**Figure 5:**
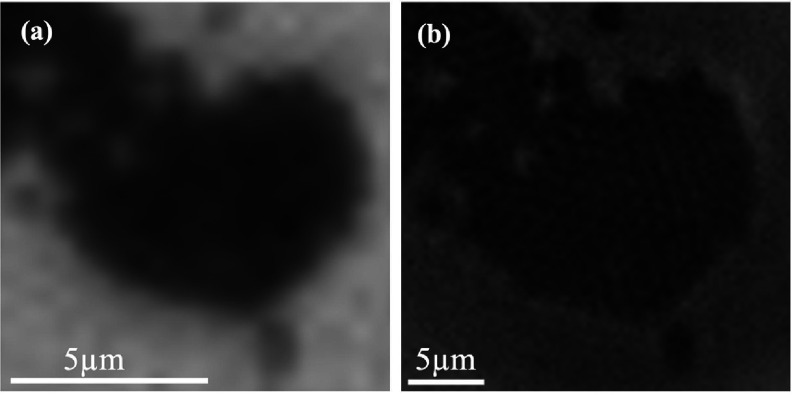
Phase contrast image of 1 *μ*m polystyrene particle acquired using (a) a 10× objective with NA of 0.25. (b) 150–180 *μ*m microlens and a 10× objective with NA of 0.25.

Following our exploration of microlens-assisted imaging technique in dark field microscopy, we also utilize phase contrast microscopy, another label-free imaging technique that is used for visualizing transparent specimens. It employs a specialized annulus and a phase plate to convert phase shifts in light passing through a specimen into variations in intensity. Figures 5(a), and (b) compares phase contrast images of 1 *μ*m polystyrene particles without and with microlens respectively, magnified 3×–4× and rendered clearly distinguishable with the use of a microlens. The combination of microlens and phase contrast microscopy offers a promising approach for achieving high-resolution imaging of transparent structures, with low scattering signature.

Furthermore, we also used fluorescence microscopy, which typically relies on external contrast agents like dyes, proteins, or nanoparticles for labeling, or it can utilize the sample’s intrinsic fluorescence, known as autofluorescence. In fluorescence microscopy, the specimen tagged with fluorescent markers is exposed to light of a particular wavelength, causing the fluorophore to absorb the light and emit another photon with lower energy. This emitted light is then detected by photomultiplier tubes or cameras in the microscope. The fluorescence imaging process, unlike dark field and phase contrast imaging which are transmission-based and capture only the signal using the microlens, requires both excitation and emission light to pass through the microlens for image acquisition. The utilization of an inverted configuration, along with a microlens substrate, has demonstrated its effectiveness in the improvement of image resolution through two distinct mechanisms. Firstly, the excitation light is directed through the microlens, allowing for better focusing on the sample (i.e. creation of photonic nanojets) [40]. Secondly, the isotropic fluorescence emission generated by the sample is more efficiently collected due to the close proximity of the microlens, which results in enhanced signal to background ratio (Supplementary section S5). Additionally, the enhanced focusing of the excitation light increases the photon density, which also enables faster image acquisition by reducing the exposure time.

**Figure 6:**
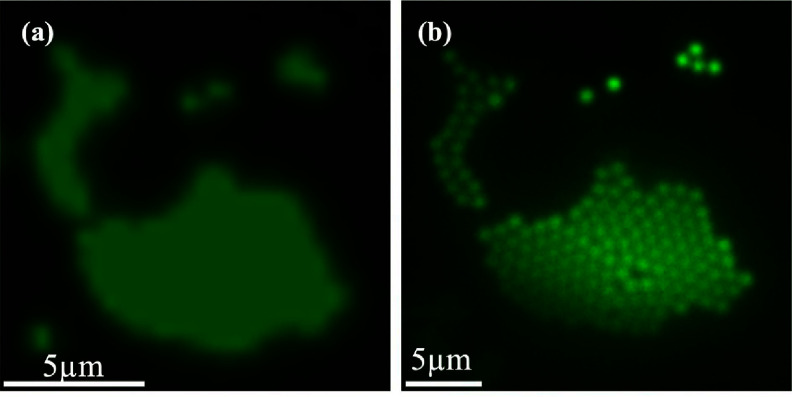
Fluorescence image of 1 *μ*m fluorescent polystyrene particles acquired using (a) a 10× objective with NA of 0.25. (b) 150–180 *μ*m microlens and a 10× objective with NA of 0.25.

We compared the fluorescence microscope images of 1 *μ*m polystyrene particles with and without microlens in figure 6. The images with the microlens-assisted technique (figure 6(b)) showed better resolution and contrast than those without microlens (figure 6(a)).

### 3.3 Imaging intracellular structures using microlens

Proof-of-concept experiments were performed to demonstrate the use of the microlens substrate for biological imaging by capturing images of fluorescent nanoparticles internalized in BT-20 breast cancer cells. Nanoparticles can enter cells through various endocytic pathways, including clathrin-mediated and caveolin-mediated endocytosis [48, 49]. Once inside the cell, nanoparticles tend to accumulate in acidic vesicles such as lysosomes [50, 51], which are involved in the degradation of cellular waste and foreign material. The acidic environment of these vesicles can cause some nanoparticles to degrade or release their cargo, which can be advantageous for drug delivery applications [52, 53]. However, this accumulation in lysosomes can also lead to potential toxicity concerns, as lysosomal damage can result in cellular dysfunction and death. Therefore, understanding the mechanisms of nanoparticle uptake and accumulation within cells is crucial for the development of safe and effective nanoparticle-based therapies especially for targeted drug delivery during cancer therapy.

**Figure 7:**
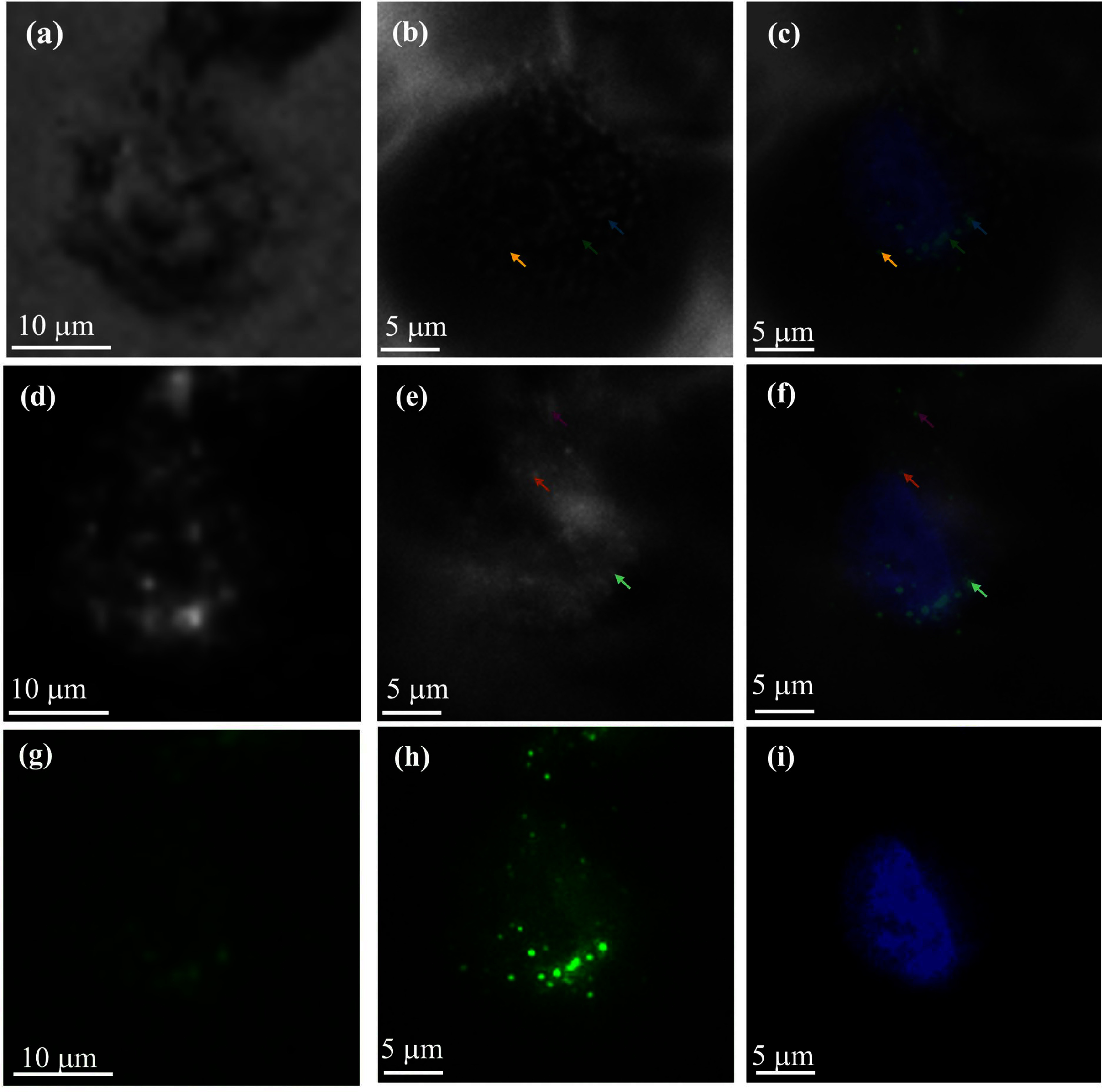
Imaging of fluorescent nanoparticle internalization within cancer cells utilizing a 10× objective with NA of 0.25 (first column), and employing a 10× objective with NA of 0.25 coupled with a 150–180 *μ*m microlens (middle column), across various imaging modalities: (a), (b) phase contrast microscopy, (d), (e) dark field microscopy, (g), (h) fluorescent microscopy in FITC channel, both images are taken in the same exposure and gain (i) microlens assisted fluorescence microscopy image of nucleus stained with DAPI. Overlays are shown for enhanced visualization: (c) phase contrast and fluorescence microscopy in FITC and DAPI channel. (f) Dark field and fluorescence microscopy in FITC and DAPI channel.

To investigate the uptake of these nanoparticles in BT-20 breast cancer cells, different imaging modalities such as dark field, phase contrast, and fluorescence microscopy with low NA objective are employed, as illustrated in figure 7. Imaging with the microlens substrate enhanced the signal to background ratio across all modalities. In phase contrast, cellular structures were more distinct. Although the dark field image (figure 7(e)) exhibited an increased background due to additional scattering from the microlens, the signal was also enhanced, thereby the signal to background ratio was not affected. In contrary, the signal increased significantly without a substantial change in background for fluorescence, even though all the images were captured with identical exposure and gain settings. A comparison of the fluorescence microscopy images in figures 7(g) and (h) demonstrates a dramatic Signal to background improvement with the microlens substrate. The arrows in the overlapping images of phase contrast and dark field microscope, combined with fluorescence microscope in figure 7, enable precise localization of cellular structures. It should be noted that while imaging with microlens substrate, precise determination of image magnification remains challenging. Therefore, we used fluorescent particles of known size (1 *μ*m) to figure out image magnification with microlens substrate.

**Figure 8:**
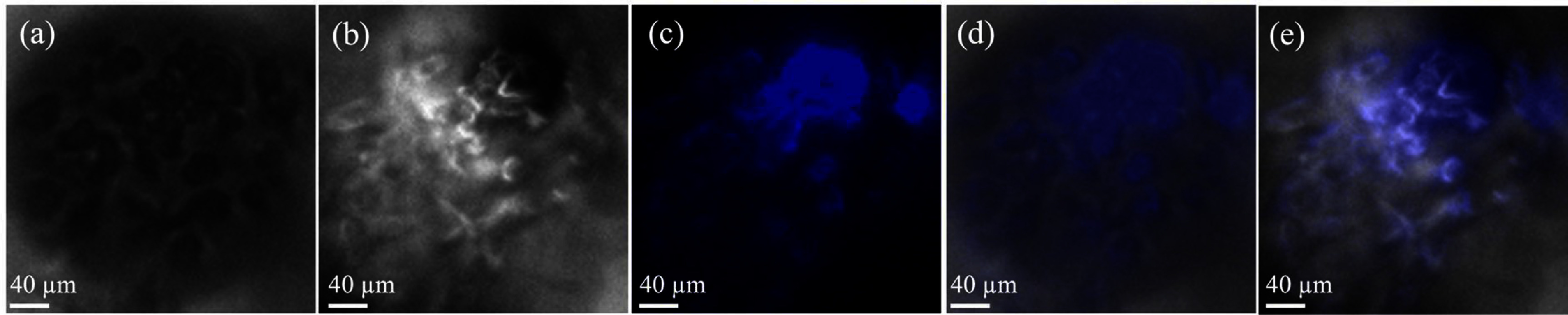
Chromosome visualization in BT-20 cells utilizing a 10× objective with an approximate NA of 0.25 in conjunction with a microlens measuring 150–180 *μ*m via (a) phase contrast microscopy (b) dark field microscopy (c) fluorescence microscopy (d) an overlay of phase contrast with fluorescence microscopy, and (e) an overlay of dark field with fluorescence microscopy.

Another proof-of-concept experiment was carried out by imaging chromosomes, which are thread-like structures found in the nucleus of cells that carry genetic information. This was performed by labeling cells with DAPI, a well-known chromosomal visualization method. As shown in figure 8, fragments of chromosomes within the nucleus, are clearly distinguishable in all the modalities. These results demonstrate the effectiveness of this method for sub-cellular imaging, showcasing its capability to resolve fine structural details within cells, thereby making it a valuable technique for exploring intricate biological processes that are not easily observed with low N.A. objectives.

### 3.4 Application of microlens-assisted mobile microscope

**Figure 9:**
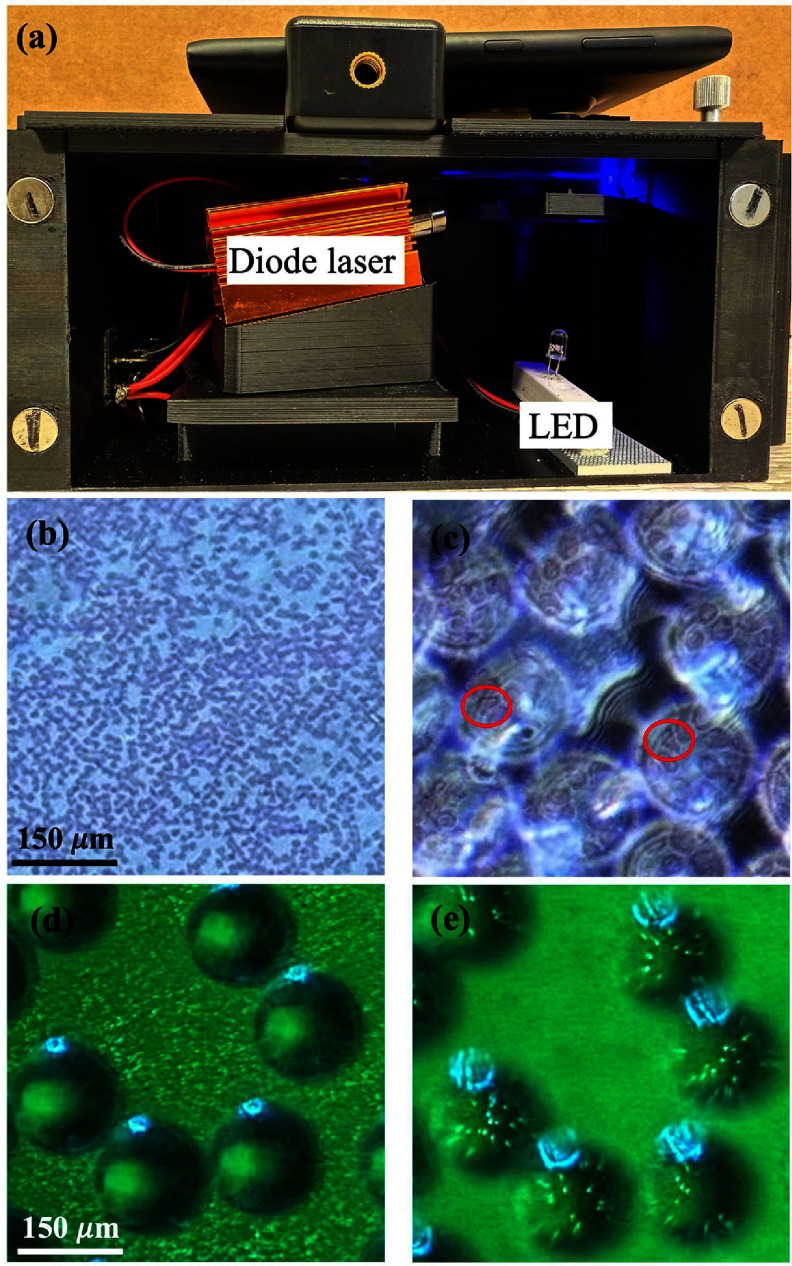
(a) Image of mobile microscope set-up. (b) Bright field images of blood smear using mobile microscope. (c) Bright field images of blood smear imaging using microlens assisted mobile microscope. (d) Imaging of 200 nm fluorescent particles while focusing on particles out of microlens. (e) Imaging of 200 nm fluorescent particles while focusing through microlens.

Microlenses enhance images captured with a low-NA objective, achieving resolution comparable to a high-NA objective. (E.g. obtaining ∼40× equivalent images using a 10× objective), without requiring any modifications to the existing microscope. This feature is particularly critical for low-cost compact microscopes used in point-of-care applications, where optical magnification is typically limited, and system configurations are rigid. To demonstrate this, we used the substrate to enhance the resolution of a mobile microscope, which is a compact and portable imaging device designed for on-site medical and scientific applications. We used the mobile microscope to perform imaging in both bright-field and fluorescence modes. As shown in figure 9(a), for bright-field imaging, the sample was illuminated from below using a white light LED. The images were then magnified with a 9 mm focal length lens and captured using a smartphone. For fluorescence imaging, the sample was illuminated using a diode laser (*λ* = 450 nm) from the side at an angle close to ∼90 degrees with respect to the camera, and an inexpensive emission filter was used to block the excitation light, prior to capturing the image using a smartphone. Proof-of-concept experiments were performed by imaging sickle cells using bright field modality. These cells are known for their distinctive morphology—a genetic disorder that causes the deformation of red blood cells (deformed RBCs). One of the standard diagnostic methods for sickle cell anaemia involves imaging a blood smear and quantifying the number and types of RBCs present. Figure 9(b) shows bright-field imaging of a blood smear using the mobile microscope (with fixed magnification), which lacks sufficient resolution to distinguish the deformed shapes of cells. However, using the microlens substrate significantly enhances the resolution, allowing clear visualization of the deformed blood cells, some of which are marked with red circles in figure 9(c). Figures 9(d) and 9) illustrate fluorescent imaging of 200 nm fluorescent particles observed at different heights. Figure 9(d) is focused on particles which are outside the microlens, and figure 9(e) shows enlarged virtual image of particles through microlens. This showcases the practical utility of our approach in medical diagnostics, particularly in resource-limited settings where conventional high-resolution microscopes may not be readily available. The ability to convert a low-cost mobile microscope, with fixed magnification, into a high-resolution imaging device using an inexpensive microlens substrate (costing <1.5 USD, under low volume manufacturing) highlights both the cost-effectiveness and ease of implementation of our approach, which can be extended to other portable microscopes as well.

## 4 Conclusion

Our work presents a novel, cost-effective, and versatile approach to enhancing the resolution of low NA, low magnification objectives using microlens-assisted techniques. Unlike traditional high NA objectives, which come with high costs, complexity, and limited accessibility, our method offers an affordable alternative that directly improves the imaging quality of widely available low NA lenses. The novelty of this work lies in its broad applicability, as the developed system is compatible with multiple imaging modalities, including phase contrast, dark-field and fluorescence imaging, and can be extended to mobile microscopy as well. This adaptability makes it highly suitable for resource-limited settings, bringing high-resolution microscopy within reach for diagnostic, educational, and research purposes. By incorporating microlens substrate in close proximity to the sample, we demonstrated sub-400 nm resolution of 1 *μ*m fluorescently labeled polymer microlens with a 10×, 0.25 NA objective across all the three imaging modalities. Through optimization, we identified the ideal microlens size and refractive index of 150–180 *μ*m and 1.48 respectively to balance resolution enhancement with field of view expansion. As a proof-of-concept, we utilized this approach for localizing intracellular nanoparticles in breast cancer cells (BT-20), which could enable a myriad of biological imaging studies. In addition, to expand our label-free imaging capabilities, we used dark field and phase contrast modalities to visualize chromosomes, an important subcellular characteristic. Beyond advancing fundamental understanding of microlens lens imaging, our work lays the foundation for affordable, high-performance portable microscopes to bring super-resolution capabilities to widespread applications in low-resource environments. By optimizing and validating microlens-enhanced imaging for robust, accessible nanoscale visualization across samples and techniques, this paper provides both a strategic blueprint and springboard for next-generation microscopy development, allowing worldwide access to powerful imaging technologies that can accelerate impactful research.

## Data Availability

All data that support the findings of this study are included within the article (and any supplementary files).

## References

[1] Masters B R, Masters B R (2020). Abbe’s Theory of Image Formation in the Microscope Superresolution Optical Microscopy: The Quest for Enhanced Resolution and Contrast.

[2] Piston D W (1998). Choosing objective lenses: the importance of numerical aperture and magnification in digital optical microscopy. Biol. Bull..

[3] Luo W, Zhang Y, Feizi A, Göröcs Z, Ozcan A (2016). Pixel super-resolution using wavelength scanning. Light: Sci. Appl..

[4] Defienne H, Cameron P, Ndagano B, Lyons A, Reichert M, Zhao J, Harvey A R, Charbon E, Fleischer J W, Faccio D (2022). Pixel super-resolution with spatially entangled photons. Nat. Commun..

[5] Wu Y, Zhang Y, Luo W, Ozcan A (2016). Demosaiced pixel super-resolution for multiplexed holographic color imaging. Sci. Rep..

[6] Luo Z, Yurt A, Stahl R, Lambrechts A, Reumers V, Braeken D, Lagae L (2019). Pixel super-resolution for lens-free holographic microscopy using deep learning neural networks. Opt. Express.

[7] Zheng G, Shen C, Jiang S, Song P, Yang C (2021). Concept, implementations and applications of Fourier ptychography. Nat. Rev. Phys..

[8] Konda P C, Loetgering L, Zhou K C, Xu S, Harvey A R, Horstmeyer R (2020). Fourier ptychography: current applications and future promises. Opt. Express.

[9] Zheng G, Horstmeyer R, Yang C (2013). Wide-field, high-resolution Fourier ptychographic microscopy. Nat. Photon..

[10] Ou X, Horstmeyer R, Zheng G, Yang C (2015). High numerical aperture Fourier ptychography: principle, implementation and characterization. Opt. Express.

[11] Heintzmann R, Huser T (2017). Super-resolution structured illumination microscopy. Chem. Rev..

[12] Demmerle J, Innocent C, North A J, Ball G, Müller M, Miron E, Matsuda A, Dobbie I M, Markaki Y, Schermelleh L (2017). Strategic and practical guidelines for successful structured illumination microscopy. Nat. Protocols.

[13] Saxena M, Eluru G, Gorthi S S (2015). Structured illumination microscopy. Adv. Opt. Photon..

[14] Rivenson Y, Göröcs Z, Günaydin H, Zhang Y, Wang H, Ozcan A (2017). Deep learning microscopy. Optica.

[15] Rivenson Y (2018). Deep learning enhanced mobile-phone microscopy. ACS Photonics.

[16] Darafsheh A, Limberopoulos N I, Derov J S, Walker D E, Astratov V N (2014). Advantages of microsphere-assisted super-resolution imaging technique over solid immersion lens and confocal microscopies. Appl. Phys. Lett..

[17] Xie Y, Cai D, Pan J, Zhou N, Guo X, Wang P, Tong L (2022). Chalcogenide microsphere-assisted optical super-resolution imaging. Adv. Opt. Mater..

[18] Darafsheh A, Limberopoulos N I, Derov J S, Walker D E, Astratov V N (2013). Comparison between microsphere-assisted and confocal microscopies.

[19] Upputuri P K, Pramanik M (2017). Microsphere-aided optical microscopy and its applications for super-resolution imaging. Opt. Commun..

[20] Krivitsky L A, Wang J J, Wang Z, Luk’yanchuk B (2013). Locomotion of microspheres for super-resolution imaging. Sci. Rep..

[21] Wang Z, Guo W, Li L, Luk’yanchuk B, Khan A, Liu Z, Chen Z, Hong M (2011). Optical virtual imaging at 50 nm lateral resolution with a white-light nanoscope. Nat. Commun..

[22] Lai H S S, Wang F, Li Y, Jia B, Liu L, Li W J (2016). Super-resolution real imaging in microsphere-assisted microscopy. PLoS One.

[23] Yang H, Gijs M A M (2015). Optical microscopy using a glass microsphere for metrology of sub-wavelength nanostructures. Microelectron. Eng..

[24] Darafsheh A, Walsh G F, Dal Negro L, Astratov V N (2012). Optical super-resolution by high-index liquid-immersed microspheres. Appl. Phys. Lett..

[25] Zhang T, Li P, Yu H, Wang F, Wang X, Yang T, Yang W, Li W J, Wang Y, Liu L (2020). Fabrication of flexible microlens arrays for parallel super-resolution imaging. Appl. Surf. Sci..

[26] Huszka G, Gijs M A M (2018). Turning a normal microscope into a super-resolution instrument using a scanning microlens array. Sci. Rep..

[27] Li L, Guo W, Yan Y, Lee S, Wang T (2013). Label-free super-resolution imaging of adenoviruses by submerged microsphere optical nanoscopy. Light. Sci. Appl..

[28] Darafsheh A, Guardiola C, Nihalani D, Lee D, Finlay J C, Cárabe A (2015). Biological super-resolution imaging by using novel microsphere-embedded coverslips. Proc. SPIE.

[29] Brettin A, McGinnis C L, Blanchette K F, Nesmelov Y E, Limberopoulos N I, Walker D E, Urbas A M, Astratov V N 2017 Quantification of resolution in microspherical nanoscopy with biological objects.

[30] Yang H, Moullan N, Auwerx J, Gijs M A M (2014). Super-resolution biological microscopy using virtual imaging by a microsphere nanoscope. Small.

[31] Perrin S, Li H, Badu K, Comparon T, Quaranta G, Messaddeq N, Lemercier N, Montgomery P, Vonesch J-L, Lecler S (2019). Transmission microsphere-assisted dark-field microscopy. Phys. Status Solidi.

[32] Nath P, Ray A (2022). Highly fluorescent nanoparticles with perovskite core for tumor imaging. Proc. SPIE.

[33] Nath P, Patrone M, Ray A (2024). Multishell nanophotonic particles with perovskite core for ratiometric biochemical sensing and imaging. ACS Appl. Opt. Mater..

[34] So S, Kim M, Lee D, Nguyen D M, Rho J (2018). Overcoming diffraction limit: from microscopy to nanoscopy. Appl. Spectrosc. Rev..

[35] Verdaasdonk J S, Stephens A D, Haase J, Bloom K (2014). Bending the rules: widefield microscopy and the Abbe limit of resolution. J. Cell. Physiol..

[36] Li H, Song W, Zhao Y, Cao Q, Wen A (2021). Optical trapping, sensing, and imaging by photonic nanojets. Photonics.

[37] Duan Y, Barbastathis G, Zhang B (2013). Classical imaging theory of a microlens with super-resolution. Opt. Lett..

[38] Chen L, Zhou Y, Li Y, Hong M (2019). Microsphere enhanced optical imaging and patterning: from physics to applications. Appl. Phys. Rev..

[39] Guo M, Ye Y-H, Hou J, Du B (2016). Size-dependent optical imaging properties of high-index immersed microsphere lens. Appl. Phys. B.

[40] Yang H, Trouillon R, Huszka G, Gijs M A M (2016). Super-resolution imaging of a dielectric microsphere is governed by the waist of its photonic nanojet. Nano Lett..

[41] Trukhova A, Pavlova M, Sinitsyna O, Yaminsky I (2022). Microlens-assisted microscopy for biology and medicine. J. Biophoton..

[42] Khoubafarin S, Nath P, Popofski H, Ray A (2023). Two-dimensional microlens array for low-cost high-resolution bio-imaging. Proc. SPIE.

[43] Khoubafarin S, Kharel A, Malla S, Nath P, Irving R E, Kaur D, Tiwari A K, Ray A (2023). Label-free identification of cell death mechanism using scattering-based microscopy and deep learning. J. Appl. Phys..

[44] Huh S, Kanade T, Salinesi C, Norrie M C, Pastor Ó (2013). Apoptosis detection for non-adherent cells in time-lapse phase contrast microscopy. Advanced Information Systems Engineering Lecture Notes in Computer Science.

[45] Feng H, Wang X, Xu Z, Zhang X, Gao Y (2018). Super-resolution fluorescence microscopy for single cell imaging.

[46] Khoubafarin S, Kharel A, Malla S, Nath P, Kaur D, Tiwari A K, Ray A (2022). Monitoring the efficacy of chemotherapeutic drugs using dark field imaging. Proc. SPIE.

[47] Reinhardt S C M (2023). Ångström-resolution fluorescence microscopy. Nature.

[48] Karamchand L, Kim G, Wang S, Hah H J, Ray A, Jiddou R, Lee Y-E K, Philbert M A, Kopelman R (2013). Modulation of hydrogel nanoparticle intracellular trafficking by multivalent surface engineering with tumor targeting peptide. Nanoscale.

[49] Manzanares D, Ceña V (2020). Endocytosis: the nanoparticle and submicron nanocompounds gateway into the cell. Pharmaceutics.

[50] Ray A, Koo Lee Y-E, Epstein T, Kim G, Kopelman R (2011). Two-photon nano-PEBBLE sensors: subcellular pH measurements. Analyst.

[51] Ray A, Lee Y-E K, Kim G, Kopelman R (2012). Two-photon fluorescence imaging super-enhanced by multishell nanophotonic particles, with application to subcellular pH. Small.

[52] Gao W, Chan J M, Farokhzad O C (2010). pH-responsive nanoparticles for drug delivery. Mol. Pharm..

[53] Shirakura T, Kelson T J, Ray A, Malyarenko A E, Kopelman R (2014). Hydrogel nanoparticles with thermally controlled drug release. ACS Macro Lett..

